# Chondroblastoma of mandibular condyle: Case report and literature review

**DOI:** 10.1515/med-2021-0352

**Published:** 2021-09-13

**Authors:** Xiaoqin Yang, Manyi Wang, Wenfeng Gao, Di Wan, Junfa Zheng, Zhaoqiang Zhang

**Affiliations:** Department of Oral and Maxillofacial Surgery, Stomatological Hospital, Southern Medical University, Guangzhou 510280, China; Department of Oral and Maxillofacial Surgery, West China Hospital of Stomatology, Sichuan University, Chengdu, China

**Keywords:** chondroblastoma, mandibular condyle, treatment

## Abstract

Chondroblastoma is one of the uncommon benign bone tumors, particularly when located in the mandibular condyle. Such a location makes its diagnosis difficult when based on only its clinical presentation and radiographic features. Herein the current report presents a case of chondroblastoma of the mandibular condyle: its clinical presentation, radiographic features, and immediate condylar reconstruction after resection. Additionally, the relevant literature is discussed to provide clinical recommendations for its diagnosis and treatment. Chondroblastoma has been reported so infrequently in the temporomandibular joint (TMJ), more common entities should first be considered in the differential diagnosis of masses in this location. Osteochondroma is the most frequent bone neoplasm in the TMJ. Since a correct diagnosis is difficult, additional tools, such as magnetic resonance imaging (MRI) and immunohistochemical analyses, should be used for diagnostics and surgical planning.

## 1 Introduction

Chondroblastoma is a rare tumor of the bone, typically occurring at the epiphyses of long bones in the immature skeleton and accounting for less than 1% of primary bone tumors [1,2]. Primary bone tumors of the craniofacial bone are not common, comprising 2% of all primary bone tumors [3]. Chondroblastomas that arise in the craniofacial bone, particularly in the mandibular condyle, are rarer than other bones [1,4,5,6,7,8]. The current treatment recommendation is en bloc excision with preservation of important neurovascular structures [9,10]. However, condylectomy without condylar reconstruction may cause a lateral open bite on the contralateral side. Therefore, simultaneous condylar reconstruction is necessary and is recommended by most surgeons [11].

Herein the authors present a case of chondroblastoma located in the mandibular condyle for which the patient underwent immediate condylar reconstruction after resection. This article will discuss the clinical presentation, radiographic features, histological analysis, surgical treatment, and long-term follow-up for this case.

## 2 Case report

**Figure 1:**
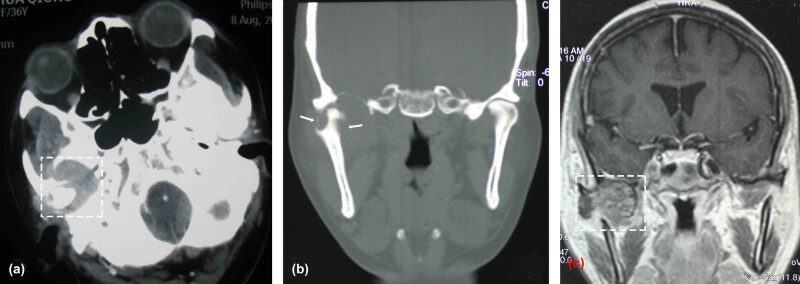
The CT scan shows a 3.5 cm^2^ × 4.0 cm^2^ expansive neoplasm located in the right TMJ region (a, horizontal plane, white rectangular region), with an expansile osteolytic process that was eroding the mandibular condyle (b, coronal plan, white arrows). MRI results confirmed an irregular neoplasm surrounding the mandibular condyle with an unclear joint space (c, coronal plan, white rectangular region).

A 36-year-old Chinese female presented with a 10-month history of limited mouth opening (30 mm), tender swelling in the right TMJ area, progressive hearing impairment in the right ear, and slight crepitation on auscultation of the right TMJ. A physical examination revealed a round, subcutaneous mass with clear boundaries located on the right preauricular site; the mass was approximately 30 mm in diameter, moderately hard, immobile, and painful with condylar movement. There was no abnormal occlusal relationship or facial paralysis. Hematological and biochemical examinations were normal. A computed tomography (CT) scan showed a 3.5 cm^2^ × 4.0 cm^2^ expansive neoplasm located in the right TMJ region (Figure 1a, horizontal plane, white rectangular region), with an expansile osteolytic process that was eroding the mandibular condyle (Figure 1b, coronal plan, white arrows) and extending into the middle cranial fossa. MRI results confirmed an irregular neoplasm surrounding the mandibular condyle with an unclear joint space (Figure 1c, coronal plan, white rectangular region). Moreover, T1- and T2-elongated signal areas in the right mastoid were indicative of inflammation. There was no evidence of either dural or intracranial involvement, based on the CT and MRI results. Ultrasonography revealed a hypoechoic mass in the TMJ region with vague boundaries and poor visualization of the bloodstream. Based on clinical features and radiographic findings prior to surgery, several diagnoses were considered: a malignant lesion, such as a chondrosarcoma, osteosarcoma, or malignant fibrohistiocytoma; and a noninvasive lesion, such as a giant cell tumor, enchondroma, eosinophilic granuloma, or aneurysmal bone cyst.

**Figure 2:**
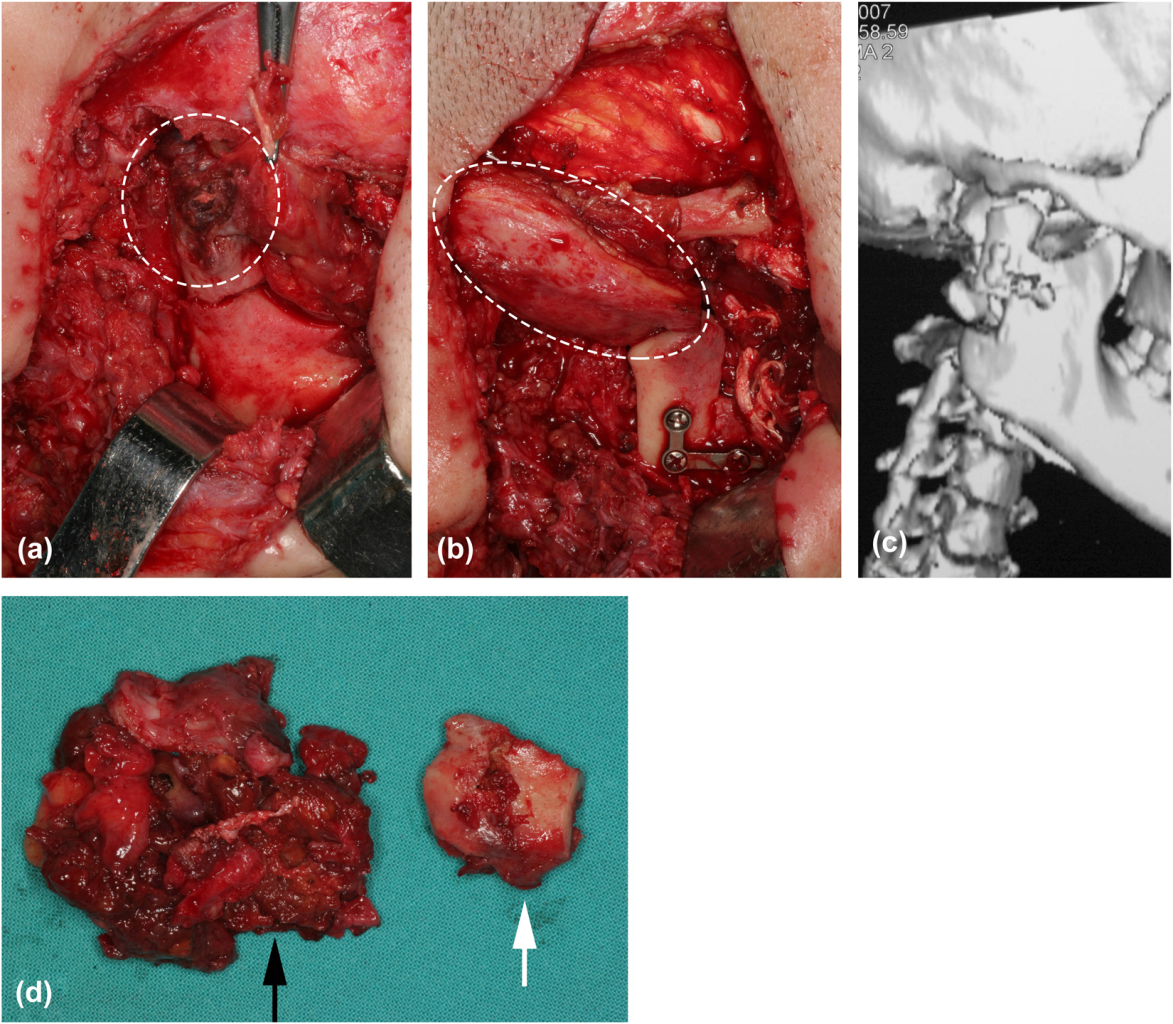
The mandibular condyle and disc were surrounded and being eroded by the neoplasm (a, white dashed region). A reverse L-shaped osteotomy line was drawn at the posterior part of the ramus. The proximal bone segment was then moved superiorly to reconstruct the mandibular condyle and fixed using an L-shaped miniplate (b, white dashed region shows temporal muscle). (c) A 3D reconstruction of postoperative CT images show the reconstructed mandibular condyle. (d) Depicts the neoplasm (black arrow) and damaged mandibular condyle (white arrow).

After preparation of the patient for surgery, excision of the lesion was carried out using a preauricular approach under general anesthesia. During surgery, a dark red neoplasm, measuring 4 cm in diameter with a hard but elastic consistency, was found. Additionally, the mandibular condyle and disc were surrounded and being eroded by the neoplasm (Figure 2a, white dashed region). Prior to surgery, histological examination of a frozen section was made on a small fragment of the mass, which revealed a low-grade malignant neoplasm. Subsequently, complete excision of the neoplasm and reconstruction of the mandibular condyle and disc were carried out.

**Figure 3:**
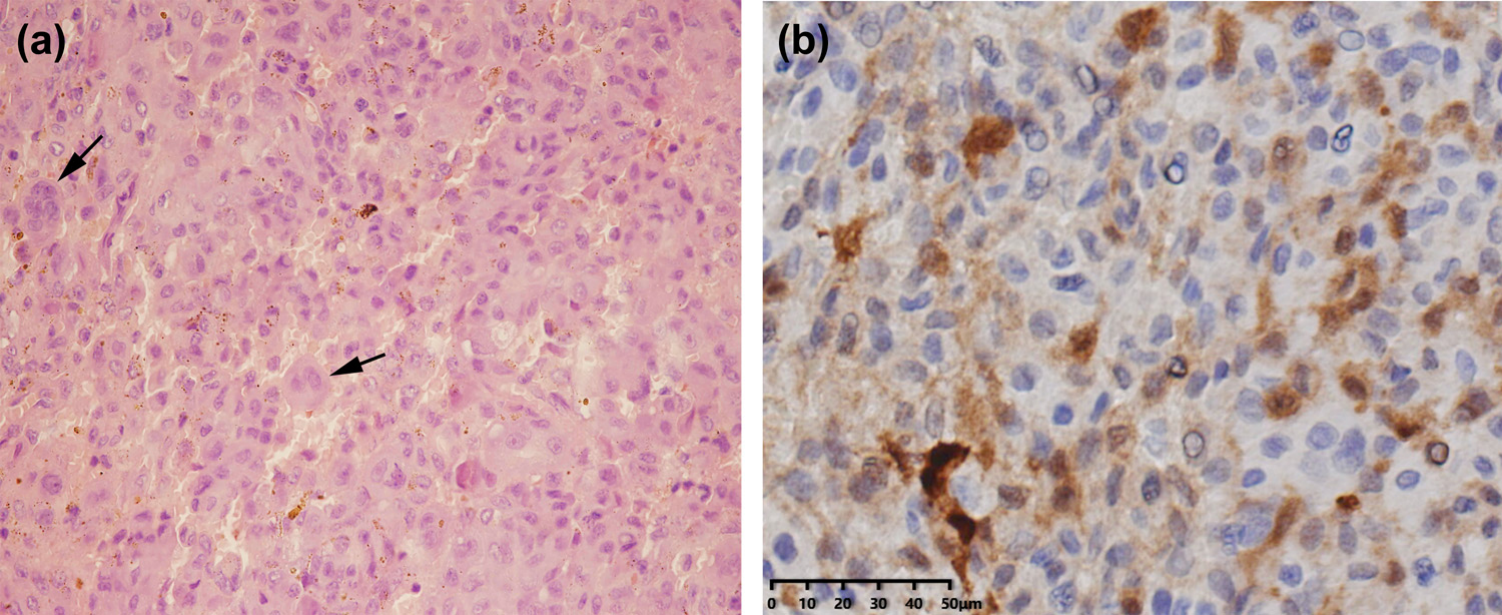
Histologically, the lesion consisted of both polygonal and round mononuclear chondroblasts with grooved nuclei as well as multinucleated giant cells (a, black arrows) in an eosinophilic cartilaginous matrix. An immunohistochemical stain for S-100 protein was positive in the tumor cells (b).

The reconstruction technique was the same as we have described in previous literatures [11,12]. Before surgery, the detailed cephalometric analysis and dental model evaluation were performed to fabricate an individual acrylic surgical splint. The splint was fixed between the maxilla and mandible in order to establish a new stable occlusal relationship. A reverse L-shaped osteotomy line was drawn at the posterior part of the ramus. Moreover, the final position of the proximal bone segment in the glenoid fossa was determined by the position of the ramus after the splint was fixed. The proximal bone segment was then moved superiorly to reconstruct the mandibular condyle and fixed using an L-shaped miniplate, while the temporalis myofascial flap was reversed anteroinferiorly to fill into the space. (Figure 2b, white dashed region shows temporal muscle). A 3D reconstruction of postoperative CT images show the reconstructed mandibular condyle (Figure 2c). Figure 2d depicts the neoplasm (black arrow) and damaged mandibular condyle (white arrow). Histologically, the lesion consisted of both polygonal and round mononuclear chondroblasts with grooved nuclei as well as multinucleated giant cells (Figure 3a, black arrows) in an eosinophilic cartilaginous matrix. Only rare mitotic activity was observed. An immunohistochemical stain for S-100 protein was positive in the tumor cells (Figure 3b), suggesting their chondroid differentiation. Based on these findings, the lesion was finally diagnosed as a chondroblastoma of the mandibular condyle. After surgery, the patient recovered hearing in the right ear. A 5-year follow-up showed no signs of recurrence, and the patient was satisfied with the function of the TMJ and was free of pain when opening her mouth.

The patient provided an informed consent to the whole treatment procedure. Further, informed consent was obtained from the patient for the publication of this case report and any accompanying images.

## 3 Discussion

Chondroblastoma is an unusual benign neoplasm of the bone. It was first reported by Codman in 1931 as a chondromatous giant cell tumor and was renamed benign chondroblastoma by Jaffe and Lichtenstein in 1942 [6,13]. Classic chondroblastomas occur at the ends of long bones, including the proximal tibia, proximal humerus, and distal femur, in children and young adults in their second decade of life, with a male:female ratio of approximately 2:1 [9]. In contrast, chondroblastomas of the craniofacial bone generally occur in older patients with an average of 47.8 years. Chondroblastoma of the craniofacial bone was first reported by Denko and Krauel in 1955 [14]. To date, approximately 60 cases of chondroblastoma of the craniofacial bone have been reported [15]. To date, only 13 cases of chondroblastoma in TMJ have been reported in English literature, of which two involved extraosseous lesions without bone invasion [9,16]. Lesions that occur at the temporomandibular joint can exhibit symptoms of swelling, and other symptoms are similar to those associated with other temporomandibular disorders, such as progressive functional restrictions, including focal pain, joint sound, and limited mouth opening, are the most important warning symptom [17,18], but in this case, facial swelling and hearing impairment were more prominent.

The radiological appearance of chondroblastoma has been described as an osteolytic process with sharply defined sclerotic margins on radiographs. In this case, the enhanced CT scan findings revealed an irregularly lobulated expansile mass with bony destruction. Although commonly reported in the literature [19], calcifications were not evident in the current case. The MRI features, however, are controversial. Flowers et al. reported a chondroblastoma of the skull in which the tumor showed hypointensity on T1-weighted images, hyperintensity on T2-weighted images, and marked contrast enhancement [19]. Kutz et al. [15]. described intermediate signal intensity on T1-weighted images and marked hyperintensity on T2-weighted images in their series. Jee et al. [20]. reported that the solid components of chondroblastomas in long bones usually reveal hypointensities on T2-weighted images, which were attributed to abundant levels of immature chondroid matrix, hemosiderin, and calcifications that were microscopically evident. The features of the tumor in the present case were similar to these previously mentioned observations.

A histopathological examination of chondroblastoma is generally characterized by sheets of polygonal, mononucleated, and neoplastic chondroblasts with thick and sharply defined cell membranes, eosinophilic cytoplasm, and round-to-indented nuclei. Interspersed within the chondroblasts are scattered collections of osteoclast-type giant cells. Also, chondroblastoma typically presents with a pink chondroid matrix and shows a distinctive pericellular deposition of calcium, which creates a “chicken-wire” formation [21]. There was sparse mitosis in this present case, but in some cases of chondroblastoma, high mitotic activity is found.

Chondroblastoma of the bone is presumed to arise from the epiphyseal cartilage prior to complete ossification because of its usual presentation in the second decade [1]. However, Spahr et al. [4] suggested that it originates from the articular hyaline cartilage, because it is anatomically attached to the articular surface of the mandibular condyle, confined to the TMJ region, and arises after the second decade.

The treatment strategies for chondroblastoma in previous cases included total en bloc excision, curettage, irradiation, and surgery combined with radiation. The most effective modality appears to be total excision with or without radiation [9]. The conservative treatment of tumors invading the temporomandibular joint using curettage alone results in a recurrence rate >55% [22]. Radiotherapy must be restricted to incomplete surgical excisions because postradiation sarcoma has been reported previously [23]. There are no reports of metastatic chondroblastoma in craniofacial bones, although there was one case of pulmonary metastasis from a noncraniofacial bone chondroblastoma [1]. Currently, chemotherapy is not a recommended treatment for chondroblastoma. A review of the literature reveals that patients with craniofacial bone chondroblastoma treated with curettage develop unacceptably high rates of recurrent disease that require secondary procedures [9]. Patients who instead undergo either en bloc excision or total resection of the tumor show no evidence of recurrence at an average follow-up of 12 months [9]. The current recommendation for treatment of chondroblastoma is en bloc excision with preservation of important neurovascular structures. Condylar reconstruction can be achieved by Transport Disc Distraction Osteogenesis, costochondral graft, a local pedicled bone graft, rib graft, sternoclavicular graft, or a custom-fitted total joint prosthesis. At present, autogenous costochondral graft is the most commonly used for TMJ reconstruction; however, this method led to some inevitable disadvantages, such as donor site deformity, the second surgical site exploration, and bone resorption [24]. A local pedicled bone graft attached to the medial pterygoid muscle formed by vertical ramus osteotomy is the most commonly used approach in our center because it avoids the second surgical site exploration and donor site deformity, and reduces the bone necrosis or resorption [11,12]. In this case, the proximal bone segment was attached by the medial pterygoid muscle, and this method could improve the stability of bone graft. Moreover, to avoid malocclusion after condylar resection, the mandibular condyle was reconstructed by the superior advancement of the posterior segment of the ramus. In this way, stable occlusion, satisfactory TMJ function, and elimination of pain on mouth opening were observed, and mandibular deviations were alleviated during the follow-up. However, further studies with more cases and longer follow-up period are necessary.

## 4 Conclusion

Chondroblastoma has been reported so infrequently in the TMJ, more common entities should first be considered in the differential diagnosis of masses in this location. Osteochondroma is the most frequent bone neoplasm in the TMJ. Chondroblastoma is often misdiagnosed as granulation tissue or a giant cell tumor. Additional tools, such as MRI and immunohistochemical analyses, should be used for diagnostic and surgical planning. Because of its high recurrence after surgical curettage, complete surgical excision and long-term follow-up are recommended as the treatment for chondroblastoma.

## Abbreviations

TMJ: temporomandibular joint
CT: computed tomography
MRI: magnetic resonance imaging
